# Transboundary Basins Need More Attention: Anthropogenic Impacts on Land Cover Changes in Aras River Basin, Monitoring and Prediction

**DOI:** 10.3390/rs12203329

**Published:** 2020-10-13

**Authors:** Sajad Khoshnoodmotlagh, Jochem Verrelst, Alireza Daneshi, Mohsen Mirzaei, Hossein Azadi, Mohammad Haghighi, Masoud Hatamimanesh, Safar Marofi

**Affiliations:** 1 Department of Watershed Management Sciences and Engineering, Gorgan University of Agricultural Sciences and Natural Resources, Gorgan 49138-15739, Iran; 2 Image Processing Laboratory (IPL), Parc Científic, Universitat de València, 46980 Paterna, Spain; 3 Department of Environment, Tarbiat Modares University, Tehran 14115-111, Iran; 4 Department of Geography, Ghent University, 9000 Gent, Belgium; 5 Research Group Climate Change and Security, Institute of Geography, University of Hamburg, 20144 Hamburg, Germany; 6 Faculty of Environmental Sciences, Czech University of Life Sciences Prague, 16500 Prague, Czech Republic; 7 Department of RS and GIS, Islamic Azad University, Tehran 14778-93855, Iran; 8 Department of Environment, Malayer University, Malayer 65719-95863, Iran; 9 Water Science Engineering Department, Bu-Ali Sina University, Hamedan 65178-38695, Iran

**Keywords:** Aras River Basin, land change modeler, remote sensing, transboundary basin, anthropogenic impacts

## Abstract

Changes in land cover (LC) can alter the basin hydrology by affecting the evaporation, infiltration, and surface and subsurface flow processes, and ultimately affect river water quantity and quality. This study aimed to monitor and predict the LC composition of a major, transboundary basin contributing to the Caspian Sea, the Aras River Basin (ARB). To this end, four LC maps of ARB corresponding to the years 1984, 2000, 2010, and 2017 were generated using Landsat satellite imagery from Armenia and the Nakhchivan Autonomous Republic. The LC gains and losses, net changes, exchanges, and the spatial trend of changes over 33 years (1984–2017) were investigated. The most important drivers of these changes and the most accurate LC transformation scenarios were identified, and a land change modeler (LCM) was applied to predict the LC change for the years 2027 and 2037. Validation results showed that LCM, with a Kappa index higher than 81%, is appropriate for predicting LC changes in the study area. The LC changes observed in the past indicate significant anthropogenic impacts on the basin, mainly by constructing new reservoir dams and expanding agriculture and urban areas, which are the major water-consuming sectors. Results show that over the past 33 years, agricultural areas have grown by more than 57% from 1984 to 2017 in the study area. Results also indicate that the given similar anthropogenic activities will keep on continuing in the ARB, and agricultural areas will increase by 2% from 2017 to 2027, and by another 1% from 2027 to 2037. Results of this study can support transboundary decision-making processes to analyze potential adverse impacts following past policies with neighboring countries that share the same water resources.

## 1 Introduction

Transboundary river basins are shared natural resources by two or more countries [1]. The United Nations Environment Program (UNEP) defines transboundary river [2] basins as river systems that pass national boundaries of countries linking them not only geographically but also politically, economically, and environmentally [3]. These rivers create hydrological, social, and economic interdependencies between societies and complicated transboundary water resource management [4]. There are 276 transboundary river basins in the world that affect 148 riparian countries with approximately 40% of the world’s population [5,6]. Managing transboundary water resources is challenging [7,8] because of the countries’ different political, cultural, development, and environmental priorities [9]. Various challenges and disputes throughout the world in the transboundary river basins have occurred because of the increasing water resources variability and consumption, demands, or claims of the riparian countries [10].

Land cover (LC) change can alter the basin hydrology by affecting evaporation, soil infiltration capacity, and surface and subsurface regimes, and ultimately affect water quantity and quality [11–13]. This appears to be especially true considering that human intrusion within this particular environment has increased in recent decades [14]. The impact of anthropogenic activities on the environment and natural resources is evident [15]. Therefore, understanding the consequences of LC changes under anthropogenic driving processes is critical for conserving biodiversity and maintaining ecosystem services [16].

A decreasing trend in water flow is globally reported [17] because of increasing water consumption. A growing global population and economic shift towards more resource-intensive consumption patterns mean that global freshwater use has increased nearly six-fold since 1900. Consequently, global freshwater consumption increased from 671.31 billion cubic meters in 1901 to 3.99 trillion cubic meters in 2014 [18]. The main drivers behind this decreasing trend are anthropogenic activities such as expanding agriculture by shrinking natural covers, developing new urban areas, using groundwater excessively, and controlling and regulating streamflow [19]. Building new reservoir dams is one of the major anthropogenic drivers of LC change that often adversely affects downstream countries in Transboundary Rivers. Although several benefits can be listed for constructing new dams, they negatively impact the environment and livelihoods by altering the natural water flow and sediment load [20,21]. Discrepancies in the needs, demands, and challenges of upstream, as opposed to downstream countries, commonly happen. Downstream countries in Asia are powerlessly exposed mainly to the negative influences of dams, whereas upstream countries unilaterally benefit from it [20].

Multiple studies have addressed the impacts of LC changes on water availability at various scales in different countries such as Yellow River basin of China [14], Goseng catchment of Indonesia [22], Heihe River basin of China [23], Zhangye city of China [24], Guishui River basin of China [25], Karst Mountain areas of China [26], Tietê water basin of Brazil [27], and Neshanic River watershed of USA [28]. Yet, only a few studies have assessed the effect of anthropogenic activities on the water in transboundary rivers [20,21,29,30].

Effective detection and monitoring of land use changes can provide accurate and timely information for land planning and management [31]. Optical remote sensing has provided a powerful tool for monitoring rapid LC changes. Recent advancements in remote sensing have equipped us with tools and technologies for analyzing land management activities [32]. In most LC change studies, Landsat images were applied because it is the only source that offers a long-term digital archive with a medium (30 m) spatial resolution and relatively consistent spectral and radiometric resolution [31]. The spatial resolution of Landsat is fine enough to monitor a variety of landscape changes [33–35].

The Aras River that flows from Turkey to the Caspian Sea along the international borders between Iran, Azerbaijan, Armenia, and the Nakhchivan Autonomous Republic is an example transboundary river that is highly affected by policies and plans especially with dam construction in the upstream countries [36]. Therefore, the main purpose of this study is to quantify LC changes over the last 33 years with an emphasis on anthropogenic activities in Armenia and the Nakhchivan Autonomous Republic. In view of predicting future LC change, potential future impacts of these changes on the water flow are also studied.

## 2 Materials and Methods

### 2.1 Study Area

**Figure 1:**
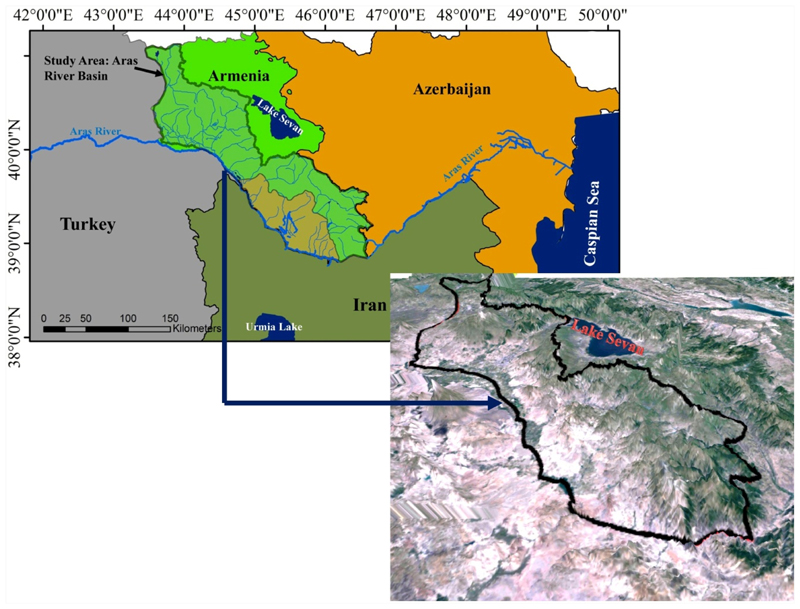
Schematic map of the study area.

The Aras River Basin (ARB) is a transboundary basin with an area of 190,110 km^2^. From its entire area, 65.4% is located in the South Caucasus countries, including 31.5% in Azerbaijan, 18.2% in Georgia, and 15.7% in Armenia (Figure 1). The remaining part belongs to Iran (19.5%) and Turkey (15.1%) [37]. The annual average precipitation over ARB is 565 mm, although it varies across the area. The annual average temperature of the entire Aras River Basin is estimated at 9 °C [37]. The Aras River has a confluence with the Kura River in Azerbaijan about 150 km before its mouth at the Caspian Sea. The Aras River originates from Turkey and forms part of the international border between Armenia and Turkey, Azerbaijan and Turkey, Armenia and Iran, and Azerbaijan and Iran [37]. The Aras River is about 1072 km long and discharges about 0.21 km^3^/year of water to the Caspian Sea, on average [38]. Aras is a vital river in the middle-east that supplies water for tens of cities and villages, thousands of hectares of cultivated land, aquaculture projects, and numerous industries [39].

Along with national projects in ARB, a few joint international projects established, mostly between Turkey and Armenia, Iran and Azerbaijan, and Iran and Armenia. For instance, a run-off-the-river hydropower project is under investigation and negotiation between Iran and Armenia [36]. Accordingly, the river water quality and quantity expected to be altered from natural flows and therefore cause changes in the LC.

This study aims to investigate LC change of the Aras River in the territory of Armenia and the Nakhchivan Autonomous Republic. In the political divisions of Armenia, the study area included the provinces of Ararat, Syunik, Vayost Dzor, Armavir, Kotayl, Aragatsotn, and Shirak, and in the political divisions of Azerbaijan, the whole Nakhchivan Autonomous Republic also studied (see Figure 1).

### 2.2 Overview of Data Collection and Research Methods

**Figure 2:**
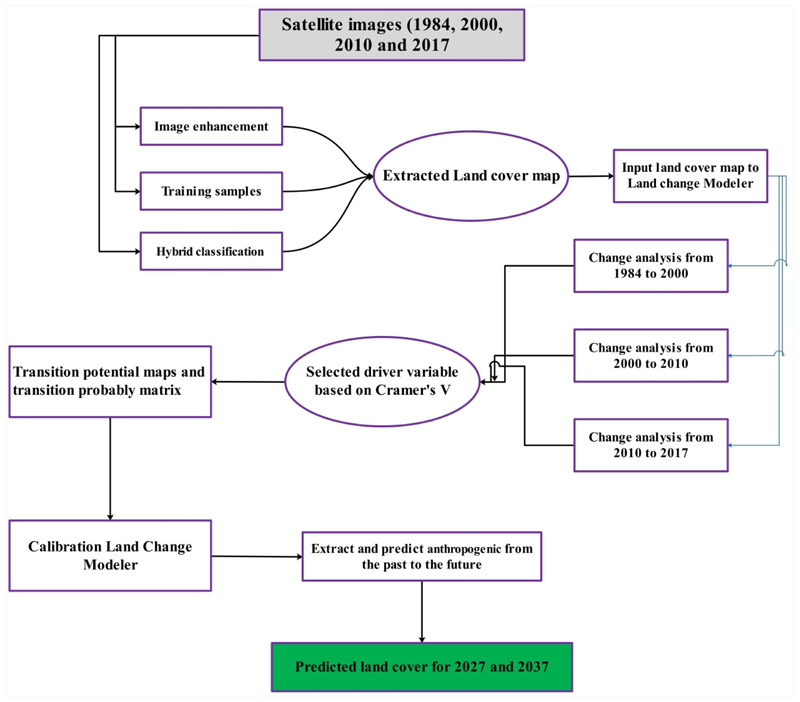
Flowchart of the research methodology.

The main research questions that this study is trying to address are: (1) Whether land class modeling can accurately simulate LC changes in ARB, (2) given that similar anthropogenic activities occur in the future, how would the LC change over the next two decades be. To answer these questions, the following five-step research is conducted: (1) Preparing satellite images, (2) producing LC maps, (3) determining transition area matrix and simulating LC maps, (4) predicting LC map in the future using the Markov Chain model, and (5) investigating future water consumption. The flowchart of the analysis process explained in Figure 2, and these steps are outlined in the following sections.

#### 2.2.1 Preparing Satellite Image and Preprocessing

Landsat 5 (TM), 7 (ETM), and 8 (OLI) satellite imageries (Collection 1, Level-1) in 1984, 2000, 2010, and 2017 were acquired from Earth Explorer website (https://earthexplorer.usgs.gov/). It should be noted that the coordinate system used in the entire image data in this study is UTM, and all of these images are projected to WGS_1984_UTM_Zone_38N. As shown in Table 1, four windows of Landsat satellite imagery applied at each studied period (with 168-33, 169-32, 169-33, and 170-32 patch-row codes) and mosaicked for covering the study area. To reduce the impacts of seasonal and phenological variation, the image pairs were selected in the same season or nearly the same season timing for all the studied years (Table 1). Therefore, temporal ranges for most scenes were restricted to within about one month. Land Cloud Cover (less than 5%) was also a critical criterion in selecting the images.

To satisfy the preprocessing requirement for change detection, multitemporal image registration, radiometric, and atmospheric corrections were accomplished first. For all scenes, preprocessing included checking geometric accuracy (minor modifications were necessary for them), as well as data stacking to place all scenes in a single composite dataset. Radiometric normalization, as well as atmospheric correction, was conducted by converting digital number to at the top of atmospheric reflectance. Finally, all scenes were stacked and used as inputs to the classifier for generating LC maps. All preprocessors were performed in TerrSet software environment.

#### 2.2.2 Producing LC Maps and Accuracy Assessment

A multi-step process involving (1) visual interpretation with true and false color composites of the images and Google Earth images [40,41], (2) images transformation maps i.e., PCA, TEXTURE, TASSCAP, NDVI, and normalized different water indices (NDWI) [42–47], and (3) supervised classification (i.e., maximum likelihood, as one of the most popular supervised classification methods used with remote sensing image data [45]) were used in IDRISI-TerrSet (version 18) software for the preparation of LC maps. It should be noted that supervised classification requires the introduction of training samples [48], so we used the visual interpretation and images transformation analysis to generate training samples. In this regard, the composite images, Google Earth images and the image transformation maps were overlapped together for identifying certain training sample in shape of small polygons. To this end, first all images were geometrically corrected based on high-resolution images of Google Earth using an image-to-image method (with an RMS error < 0.5 pixel). Then, for each of the studied LC, 180 training samples were extracted from Google Earth images. The training samples were considered the same for all the studied years, and in their selection, it was considered that the LC displayed by that point/polygon did not change over the studied period. For instance, we selected 180 training samples for the agricultural class, during which the 32-year period (1984–2016) persisted as agricultural and had not changed to other LCs. It should be noted that PCA, TEXTURE, TASSCAP, and NDVI transformation accompanied by the composites images were also used to produce these training samples for forest, agricultural, rangeland, and bare land classes [44,45].

NDWI values derived from satellite images are commonly and successfully utilized in surface water body detection and mapping (Equation (1); [49]). It is calculated as follows:

(1) NDWI=[(Green−NIR)/(Green+NIR)]

where Green is the 3rd band and NIR is the 5th band of Landsat-8 OLI. NDWI values (–1 ≤ NDWI ≤ +1) can be considered as a basis for classifying water and non-water LCs [50,51]. Also, the shallow water, which represents rivers well, can be discriminated from deep water based on the NDWI values. It is because the shallow water has higher reflectance in the green and red bands due to the bottom effect and can be classified as a separate water class [51]. To this end, NDWI threshold values can be designated both manually and automatically [51–53]. Therefore, we experimentally obtained the thresholds for separating non-water areas from shallow and deepwater classes as follows: Non-water: –1 ≤ NDWI < 0, shallow water: 0 ≤ NDWI < 0.3, and deep water 0.3 ≤ NDWI ≤ 1.

Manual digitization was also carried out to obtain higher accuracy in the classification of residential areas [54]. Finally, seven LC types i.e., (1) forest including primary forest, planted forest, woodland, and other naturally regenerated forest, (2) agricultural lands, (3) rangelands (i.e., areas that are covered by shrubland and grassland), (4) bare lands, corresponding to bare soil (i.e., areas that have no vegetation cover except water), barren areas of rock, sand, or snow with NDVI close to 0 [44], (5) deep water, (6) shallow water (river), and (7) residential areas were classified in the study area.

Ground points were used to assess the accuracy of the LC maps extracted from Google Earth high-resolution images based on simple random sampling [55]. To this end, 173, 179, 177, and 166 ground control points selected to assess the accuracy of the classification for 1984, 2000, 2010, and 2017, respectively. Finally, Kappa coefficient was used to assess the accuracy of the produced LC maps [56,57]. The Kappa coefficient can range from –1 to +1, where 1 is perfect agreement, 0 is exactly what would be expected by chance, and negative values indicate agreement less than chance [58,59].

#### 2.2.3 Land Cover Change Prediction

The land change modeler (LCM) as a part of the TerrSet software package applied to predict future LC maps for years 2027 and 2037. LCM is an integrated vertical tool to analyze LC changes and predict future LC trends [60]. Three main steps of LCM were (1) analyzing LC changes, (2) creating transition potential maps, and (3) predicting future LC maps. In the first step, LC changes were analyzed between the generated LC maps, comparing 1984 versus 2000, 2000 versus 2010, and 2010 versus 2017. In this step, LC transition maps created to analyze and determine spatial trends in LC changes as well as LC gains and losses. These maps and analyses were used to define future scenarios of LC change.

In the next step, transition potential maps were generated based on one or a group of sub-model(s) offered by LCM. In LCM, change analysis and prediction were performed by a series of empirically evaluated transition sub-models. Each sub-model simulates the conversion from one LC to another, e.g., forest to agriculture.

The difference between the sub-models is their transition potential map were defined based on scenarios of changes identified in the historical records [60]. In this study, transitions are modeled using a multi-layer perceptron (MLP) artificial neural network (ANN) [61,62]. The driving variables for each sub-model must be identified and introduced as its inputs. We identified a list of environmental variables recommended in the literature as potential drivers of LC changes [63,64]. These variables include elevation, slope, aspect, distance from roads, distance from rivers, distance from rangelands, distance from bare lands, and distance from agricultural areas. The explanatory power of these variables was tested by Cramer’s V that should have a value higher than 0.15 for influential variables [65].

Finally, in the third step, the amount of LC change that expected to occur in the future was predicted using the Markov Chain prediction process and the best-calibrated sub-model. The Markov Chain determines the amount of changes using the earlier and later LC maps along with the date specified [66]. The predictions made for LC changes in 2027 and 2037 were based on multi-objective land allocation.

#### 2.2.4 Details of Sub-Models

In order to predict the LC changes, each LC transition must be modeled empirically, called transition potential maps [67]. In other words, this is a past trend-based model such that the rate of change is obtained from the analysis of change, which occurred during a previous period (called calibration period). Also, a transition sub-model can consist of a single land cover transition or a group of transitions that are thought to have the same underlying driver variables [60]. We used two calibration periods: 1984–2000 and 2000–2010 and attempted to identify the most important transition sub-model maps for these periods. The large number of sub-models causes data processing to be slow and the low number of sub-models also leads to data loss. The choice of the optimal number of sub-models can be based on the size of the changes that have been occurred in a region. The LCM has made it possible to choose a threshold for choosing optimal number of sub-models and users can ignore the small changes [67]. Therefore, considering the extent of the study area, we ignored LC transitions that occurred in less than 700 km^2^ (less than 3% of the study area). Based on this precondition, we obtained five sub-models for the first calibration period (1984–2000) and six sub-models for the second calibration period (2000–2010). In the first calibration period, the main transition sub-models include: (1) Forest to agriculture, (2) agriculture to rangeland, (3) bare land to rangeland, (4) rangeland to agriculture, and (5) all transmission sub-models. Furthermore, over the second calibration period, sub-models include: (1) Agriculture to forest, (2) agriculture to rangeland, (3) forest to agriculture, (4) rangeland to agriculture, (5) rangeland to bare land, and (6) all transmission sub-models. Tables 2 and 3 show the details of sub-models of the first and second calibration periods, respectively. We then used the identified sub-models to generate transmission potential maps, the so-called soft map that assigns a number from [0, 1] to each pixel to demonstrate its potential for the transition from one LC to another one. In the transition potential modeling process, sub-models can be defined by enabling or disabling identified transitions to determine modeling scenarios. These sub-models have been combined in different scenarios to obtain the best modeling results compared to historically observed LC changes. The sub-model (scenario) with the highest prediction accuracy based on the Kappa index for the LC map of 2017 has been selected to predict LC maps for 2027 and 2037.

## 3 Results

### 3.1 LC Maps

**Figure 3:**
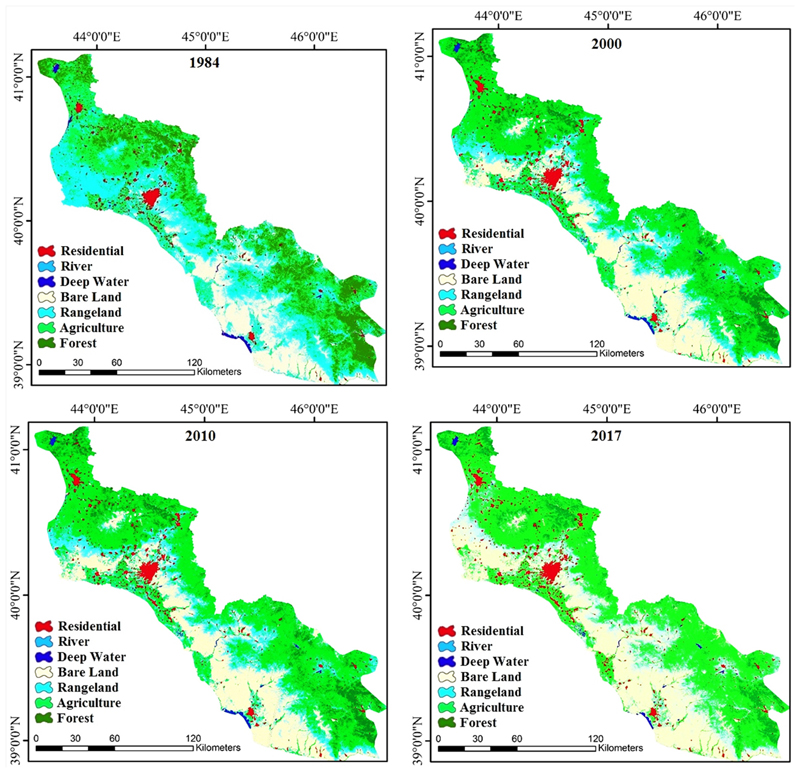
The LC classification maps for 1984, 2000, 2010, and 2017 in Aras River Basin (ARB).

The LC classification results have been obtained with the maximum likelihood method in 1984, 2000, 2010, and 2017 are shown in Figure 3. In this analysis, 173, 179, 177, and 166 ground control points selected to assess the accuracy of the classification based on the Kappa index for 1984, 2000, 2010, and 2017, respectively. Table 4 gives more details of classification accuracies for different LC classes. The most difficult LC class to distinguish over the years was agriculture that was very similar to rangeland and bare land areas. Residential areas scored a good classification result in all four periods with more than 86% classification accuracy because they distinguished by both supervised and on-screen digitize methods. The overall Kappa index was 87%, 87%, 85%, and 88% for 1984, 2000, 2010, and 2017, respectively.

### 3.2 Anthropogenic LC Changes

#### 3.2.1 Changes in Water Bodies

**Figure 4:**
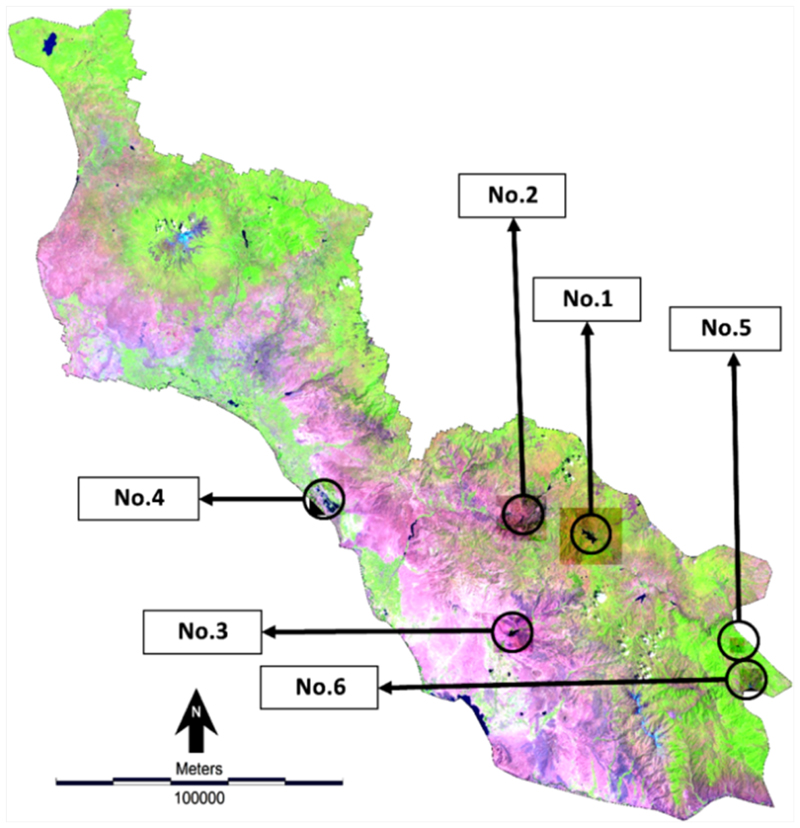
Location of Vam activities over the past 33 years.

**Figure 5:**
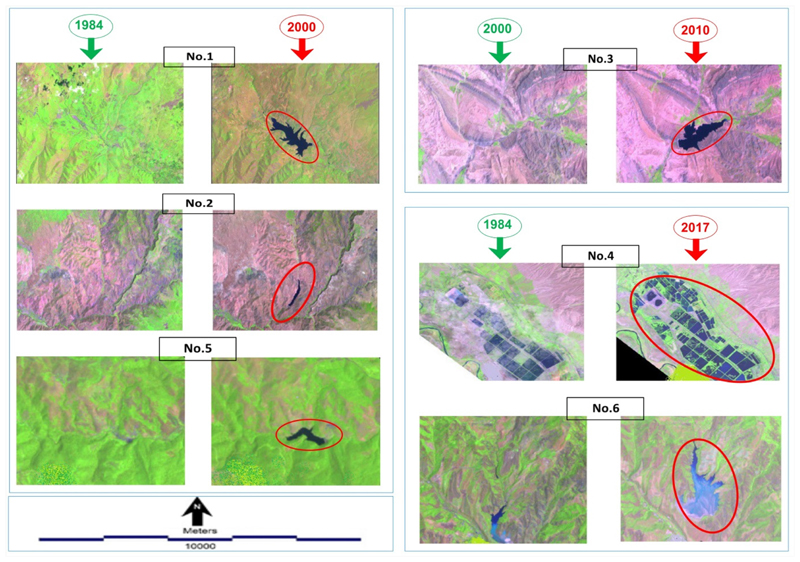
Comparing changes in water bodies by making false-color composite in Aras River basin.

The LC analysis shows that the major changes in water bodies of ARB from 1984 to 2017 include changes in the surface elevation of lakes, changes in the depth and width of rivers, and development of new lakes created by new reservoir dams. More specifically, the satellite image processing of this study identified six important changes in water bodies of ARB over the past 33 years. The geographic location of these changes shown in Figure 4, and the type and extent of them shown in Figure 5. As shown in Figure 4, dam No. 1 is located in the eastern part of the basin with the aim of water storage. It was operational in 2000 and had a lake surface area of 724.5 hectares at the time of capturing the satellite image (22/07/2000). Dam No. 2 created a lake with a surface area of 98 hectares in 2000 to supply agricultural demands. Dam No. 3 was operational in 2010 to store water for agriculture and generate hydropower in Nakhchivan and created a lake with a surface area of 283 hectares. No. 4 is the biggest construction of an aquaculture production with an area of 1403 hectares. Dams No. 5 and 6 are located in the eastern part of the basin and aim to supply water for agriculture and increase infiltration. See Table 5 for full details about these six major changes in water bodies of the study area.

**Figure 6:**
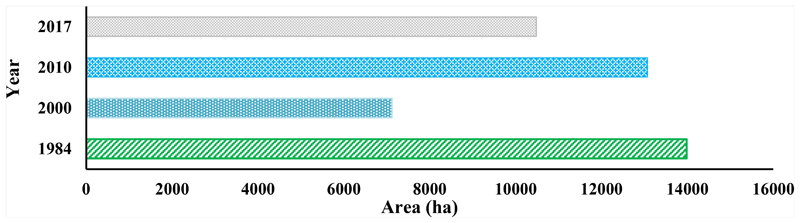
Deep water changing from the past 33 years to present in Aras River basin.

According to the extracted LC types from satellite images of 1984, 2000, 2010, and 2017, water bodies of ARB underwent some fluctuations in these periods. The most significant changes occurred from 1984 to 2000, when the area of water bodies decreased from 13,983 to 7120 hectares. A similar decreasing trend was observed from 2010 to 2017 and attributed to storing water behind the dams. On average, the decreasing rate in the area of water bodies from 1984 to 2017 is around 106 hectares per year. More details about changes in the area of water bodies are shown in Figure 6. Overall, changes in water bodies in comparison to changes in other LCs explained in the next section during the past 33 years are limited and on a small scale.

#### 3.2.2 Changes in Agriculture, Forest, and Rangeland

**Figure 7:**
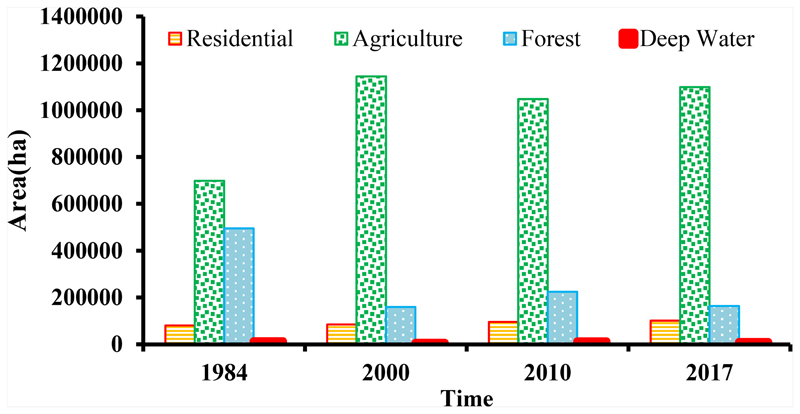
Area of LC in 1984, 2000, 2010, and 2017.

According to Figure 7, severe LC changes caused by human activities from 1984 to 2017. The area of forest that: is the most important LC for the sustainability of a basin dramatically decreased by 66.7% from 495,370 hectares in 1984 to 164,511 hectares in 2000. The majority of this LC converted to agricultural land. Moreover, in the same period, 122,280 hectares of rangelands converted to agricultural land. Another notable change in this period is the conversion of 1075 hectares of agricultural land to residential areas.

Figure 7 shows an increasing trend in the area of the residential LC in ARB. From 1984 to 2000, 4080 hectares converted to residential areas. This increasing trend accelerated and resulted in another 10,601 hectares of new residential areas from 2000 to 2010 and 5725 hectares from 2010 to 2017.

### 3.3 LC Map Processing and Validation

**Figure 8:**
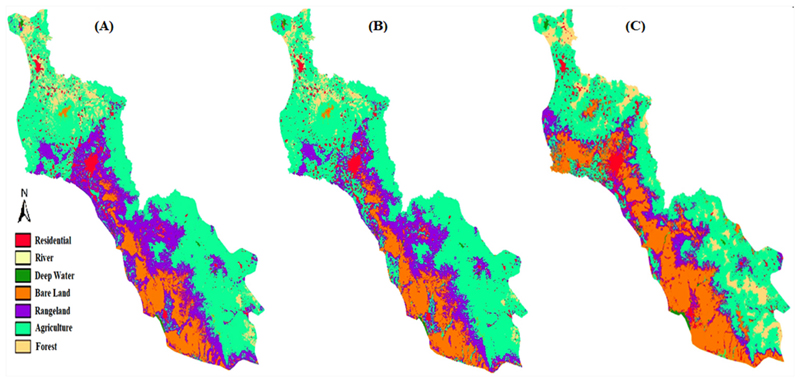
LC maps predicted for 2010 (**A**) and 2017 (**B**) based on LC changes observed from 1984 to 2000 andthe predifted LC map for 2017 (**C**) based on the 2000–2010 calibration period.

The variables listed in Table 6 considered as the most important drivers of LC changes in the study area. To achieve the best-performing LCM, various sub-models developed using different combinations of these variables. LC changes observed from 1984 to 2000 were used to calibrate the LCM model. The calibrated model showed high accuracy in the validation phase by scoring a Kappa index of 89% and 86% to predict the LC map of the study area in 2010 and 2017, respectively (Figure 8A,B). When LC changes from 2000 to 2010 are used to calibrate the model, its accuracy for predicting the LC map of 2017 dropped to 84%, (see Figure 8C). It is worth noting that rivers were classified with the lowest prediction accuracy in terms of the Kappa index. The reason for this suboptimal accuracy is attributed to the dynamic nature of rivers that changes them over a relatively short period.

### 3.4 Predicting Future LCs

**Figure 9:**
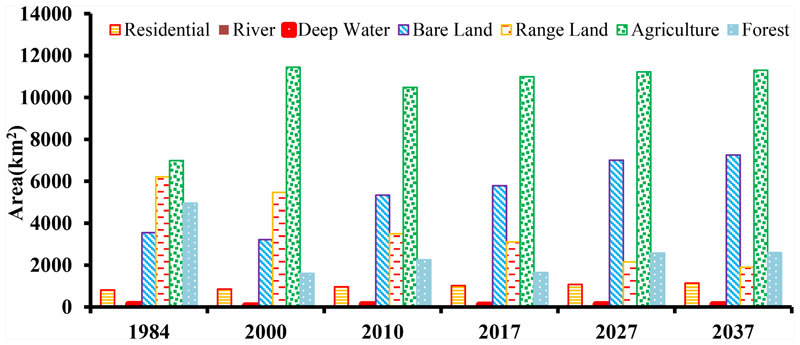
Area of major LC changes observed in ARB from 1984 to 2017 and predicted for 2027 and 2037.

**Figure 10:**
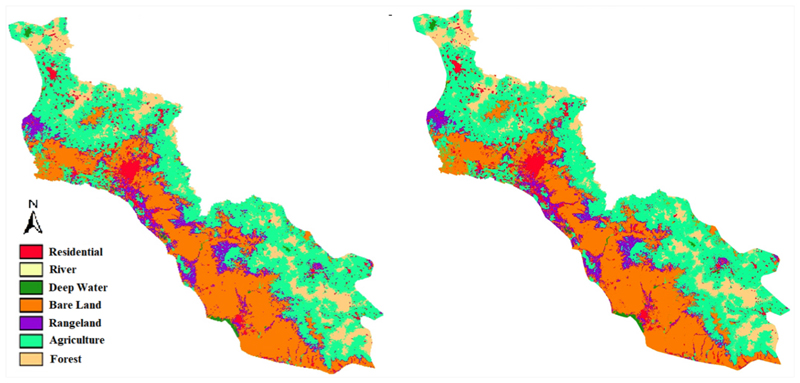
Prediction of LC changes in the basin for 2027 (**left**) and 2037 (**right**).

The most accurately calibrated LCM has been used to predict the LC maps for the years 2027 and 2037. The are as of these six classes of LC classes for all years from the past to the future were calculated and are shown in Figure 9. Moreover, the predicted LC maps for 2027 and 2037 are shown in Figure 10.

According to Figure 9, the area of rangeland was expected to decrease by 957 km^2^ and 1207 km^2^ in 2027 and 2037, respectively. On the other side, the areas of forest, bare land, agriculture, and residential area are expected to increase by 935 km^2^ and 949 km^2^, 1216 km^2^ and 1463 km^2^, 230 km^2^ and 305 km^2^, and 58 km^2^ and 125 km^2^ in 2027 and 2037, respectively.

## 4 Discussion

This research combined LCM and Markov chain prediction models to enable analyzing LC changes in both temporal and spatial dimensions. The model parameters were created based on the historical LC data of 1984, 2000, 2010, and 2017. Environmental drivers and different scenarios analyzed in order to understand the pattern of changes in LC in the area. The models were run several times in order to realize the optimal model and then LCM was used to predict changes in LC for the future. The results of the evaluation of transition potential modeling using ANN-MLP showed more than 80% accuracy in most scenarios. In the study by Pérez-Vega et al. [68], the transition potential modeling with ANN modeling was carried out. They processed three sub-model series, i.e., recovering sub-model (agricultural land to tropical deciduous forests and agricultural land to secondary tropical deciduous forests), deforestation sub-model (tropical deciduous forests to agricultural land, tropical deciduous forests to rangeland, secondary tropical deciduous forests to agricultural land, and secondary tropical forests to rangeland), and perturbation sub-model (tropical deciduous forests to secondary tropical deciduous forests). Finally, they achieved an accuracy of 59.2%, 35.2%, and 59.6%, respectively. As opposed to that study, here we achieved a higher accuracy. Our obtained high accuracy of transition potential modeling using ANN compared to the study by Pérez-Vega et al. [68] is due to some powerful significant qualitative variables in LC changes or the choice of more appropriate input variables in each sub-model in the present study. In another study, Khoi and Murayama [69] achieved similar results in modeling transition potential with an ANN (about 80% accuracy) in Tam Dao National Park. Furthermore, in a study by Oñate-Valdivieso and Bosque Sendra [70], more than 70% accuracy in all transitions was achieved using an ANN, which is similar to the results of the present study.

Since the ARB is a populated basin, the LC analysis was concentrated on anthropogenic impacts mainly on construction, residential areas, forest degradation, and agriculture. Land use change occur at the local scale, but its environmental influences spread across regional and global scales [71]. Expansion of agricultural land is the most pervasive anthropogenic land conversion process in Asia [72]. Based on the obtained results, the agriculture class has increased by 399,930 hectares from 1984 to 2017. Most agricultural lands currently in use occupied former primary forests, rangeland, and wetlands [73,74]. Agricultural activities have caused serious degradation of the soil in South and Southeast Asia [75].

In this study, substantial changes occurred in water bodies during the years 1984 to 2017, such as the decrease and increase in the water level of lakes and changes in depth and width of rivers and depositing and building new constructions. However, processing images showed six important changes in water bodies over the past 33 years, in particular the dam site. The rapid construction of dams in Asia has created wide-spreading loss or fragmentation of freshwater habitats, particularly those of riparian floodplains and wetlands, although dams have executed a significant role in water supply, flood control, irrigation, and hydroelectric power production [72].

According to the captured satellite images for these years 1984, 2000, 2010, and 2017, as well as extracting LC for these years, water bodies have some fluctuations in this period. Most changes took place in 2000 with a decrease in the area from 13,983 to 7120 hectares from 1984 to 2000. This decrease continued from 2010 to 2017. Overall, changes in water bodies in comparison to other LCs during the past 33 years are limited and on a small scale. These findings are consistent with a study by Hajihosseini et al. [76]. They quantified the causes of the decrease of streamflow in the transboundary Hirmand (Helmand) located between in in Iran and Afghanistan. Th same as the current study, they applied remote sensing to understand watershed response to environmental changes and land use change. Their finding showed that agricultural developments in the Helmand basin decreased the discharge with about 346 million cubic meters/year accompanying an increase of 64,000 ha in an irrigated area in the river. Their results also revealed that the impact of land use change increased significantly for more recent periods and causes a reduction of 810 million cubic meters in annual streamflow. In another similar study by Al-saady et al. [77], they investigated impact of land use expansion on the natural environment of the Little Zab River basin in Iraq. Using three indices, NDVI, NDWI, and difference built-up index (NDBI), the change detection of the river has been performed considering six main classes of land use land cover. Their findings showed that continuous population growth with increasing individual demand for water used for irrigation, urban, and industrial uses resulted in intense competition in the distribution of the watershed’s scarce water resources.

At the same time, it is common knowledge that LC practices such as deforestation and regeneration of wetlands also affect the quality and wellbeing of freshwater bodies. Deforestation has contributed to river siltation and failing to restore the wetlands has reduced the ability of river basins to control flows, leading to significant flooding. Both of these actions modify freshwater environments causing species to decline or vanish in them [78,79]. Moreover, forests as one of the most important LC classes to the sustainability of a basin underwent a big decrease from 1984 to 2000. Accordingly, the area of forest class decreased from 495,370 hectares in the first period to 164,511 hectares in the second period, and the change affects ~66.7 percent of the whole area of forest class in the study area. These results are in line with the results of Moreno-Sanchez et al. [80], who showed that the total area of forests in Armenia decreased from 294,135 in the period 1987–1989 to 246,098 in the period 2000–2001. Furthermore, regarding the transmission sub-models, almost all of these changes have converted to agriculture. This is in line with a general trend for the wider region. According to Houghton and Hackler [81], in most parts of Asia, forest biomass and soil carbon have been reduced by 58% and 18%, respectively. Deforestation and forest fragmentation also created significant threats to the flora and fauna that live inside [72]. Ogutu et al. [82] reported a decline in some wild animal species (e.g., giraffe, waterbuck, impala) in the pastoral ranches bordering the Maasai Mara National Reserve, largely due to gradual degradation of forests and land use change.

In this study, the residential areas have grown during the years 1984, 2000, 2010, and 2017 by 81,290, 85,370, 95,971, and 101,696 hectares, respectively. Residential development in the basin has grown, and considering the direct link between residential area development and water consumption, it implies that much more water consumed in upstream parts of the basin. LC practices such as deforestation and regeneration of wetlands also influence the nature and protection of freshwater bodies. Kiragu [83] reported high sedimentation in Mara River, which is linked to watershed deforestation and intensive agriculture currently practiced in east Africa. According to Kiragu [83] study, agricultural deforestation and intensification are likely to cause an increase in surface runoff due to watershed degradation, which decreases its ability to absorb rainwater (reduced infiltration).

**Figure 11:**
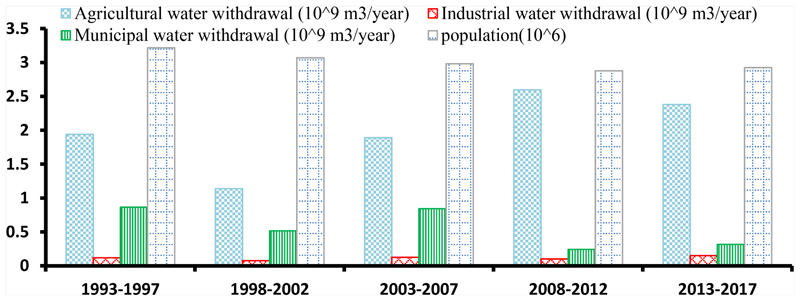
Amount of water consumption in three different LC classes and the population of Armenia.

In line with the recent trend, continuous expansion of the agricultural LC class is expected. Consequently, the growth process in agriculture will lead to increased water consumption and water demand. Meantime, the decreasing trend in rangeland class will decrease infiltration and resilience in the basin, and the volume of sedimentation will increase. Since the Aras River Basin is a transboundary basin, more water consumption could lead to environmental risk in the commonwealth, particularly in downstream areas like Iran. According to the data that were collected from the FAO [84], the amount of water consumption in three different types of LCs, including agricultural, residential, and industrial areas, over time was calculated. The population of Armenia was also collected from 1993 to 2017 based on the FAO [84] database. As shown in Figure 11, water consumption in agriculture has a significant increasing trend from 1993 to 2017, which is in good agreement by the extracted agriculture in Landsat images and its expansion; again, this trend can be concluded as industrial consumption. Also, in Armenia, land area devoted to cereals declined over the past decade, with an 18% decrease from 2001 to 2010, where land area devoted to fruits increased by 25%, and land area devoted to vegetables, including melons, increased by 21% from 2001 to 2010 [85], which indicates a change in cultivation pattern and the use of species with higher water requirements, which can be one of the reasons for increasing water harvest and consumption in agriculture. Although water consumption in agriculture and industrial areas has increased over time, based on the results, the amount of water consumption in the residential areas has decreased. This decrease can be due to a decreasing trend in population and changes encouraged by the Armenia government, such as modification of water consumption patterns. These modifications of patterns can include reducing bath time, using water-reducing appliances, identifying, repairing, or replacing damaged appliances, and so on. Also, this dramatic drop is attributed to the introduction of water metering and a volumetric billing system.

Finally, water availability in the Aras river basin is of shared interest among Armenia, Turkey, and Iran [86]. The downstream riparian countries including Armenia, Iran, Georgia, and Azerbaijan have identified the major transboundary issues in the Kura-Aras basin as being: Hydrological flow variation and reduction, water quality deterioration, river basin ecosystem degradation, and increased flooding and bank erosion [86].

Construction of dams and the expansion of irrigated farms in upstream areas would undoubtedly affect flow patterns, water availability, and freshwater ecosystems, such as wetlands and alluvial plains in the downstream states, mainly Iran and Azerbaijan [86,87].

**Figure 12:**
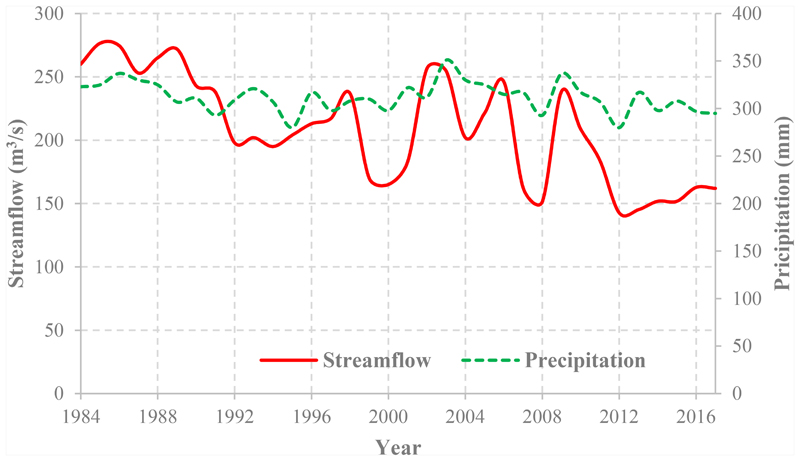
Decrease in stream flow data based on one of the hydrometric stations located on the outlet of the basin (https://irandataportal.syr.edu/ministry-of-energy).

The present study is indicative of the large influences that LC change has on water resources in the outlet of the case study as domestic end-users. Based on the data that were recorded by the Ministry of Energy in Iran, approaching 2017, the trend of the observed streamflow showed a remarkable reduction (see Figure 12), which can be attributed to increased water withdrawal because of LC change, especially agricultural activities. This finding is inconsistent with a study by Mwangi et al. [88], as they estimated that land use change in the last 50 years contributed to about 97% of the observed increase in mean streamflow of Nyangores River (a headwater tributary of the Mara River in east Africa).

Based on prediction maps for the years 2027 and 2037, as well as the results for the past, the governments of Armenia and the Nakhchivan Autonomous Republic are developing agriculture and residential areas and constructing dams. Consequently, according to the articles 5, 6, and 7 of the Convention on the Law of Non-Navigational Uses of International Watercourses Adopted by the General Assembly of the United Nations on 21 May 1997, “These activities should not violate the rights of stakeholder downstream countries”.

### Limitations of the Study

Finally, we also encountered some limitations when conducting the analysis. Different seasonal timing of Landsat images can dramatically influence the LC classification, especially in LC such as agriculture, rangeland, and bare land, which are strongly related to NDVI. In this respect, the most important limitations we faced were dealing with the incompleteness of the USGS-Archive as well as the extreme Land Cloud Cover of the study area during our chosen dates. In order to overcome this limitation in future studies, and so to better understand the conversion of rangeland to bare land, it is suggested to work with a more complete version of USGS-Archive.

## 5 Conclusions and Outlook

LC changes and modifications of the Aras River in the territory of Armenia and the Nakhchivan Autonomous Republic over 33 years from 1984 to 2017 investigated to predict LC maps in the years 2027 and 2037. Environmental driver variables and different scenarios defined to understand the pattern of changes in LC in the Aras river basin, and the models were operated several times to realize the optimal model and then, using LCM, predict changes in LC for the future. The outcomes showed that the models are able to predict the LC changes in the study area with accuracy from 81% to 85% using the Kappa index. This study focused on anthropogenic impacts mainly on construction and agriculture in the basin, and based on the outcomes, six important dams and aquacultures built on the upstream of the basin over the past 33 years. On the other hand, agriculture and residential classes as the most important water consumers have increased around 399,930 and 20,406 hectares from 1984 to 2017, respectively. In addition, according to prediction maps, the increase in this trend continues in 2027 and 2037 for these classes. It should be regarded that water supply for farming and guarantying the security of agriculture will undoubtedly be a concern for the governments of Armenia and the Nakhchivan Autonomous Republic in the Aras River Basin. For these reasons, the governments have some simple options such as constructing a dam and seal and water diversion from the main channel for more irrigation and consumption. Overall, since the basin is a transboundary basin between Iran, Turkey, Armenia, Azerbaijan, and Georgia, and considering the importance of water in political relationships, a more balanced strategy for land covers and harvesting rivers should be applied. The international development experience of transboundary territories and the results of our study demonstrate that sustainable water resource management can be realized in specific territorial trends. Under poor water resource management, one frontier territory may, in general, have a negative impact on the management of a neighboring border territory. Comparative assessments of water resource management trends that are built on the neighboring countries’ frontier territories are therefore required. At the same time, studying the long-term shifts in land use land cover change in some part of the world is one of the most critical tasks of development studies.

The study of all the complex problems affecting land use efficiency and ecological conditions in transboundary river basins, as well as the factors undermining their systemic structure, is therefore one of the primary objectives policy makers, which should be designed for all basins. The methods applied in this study can be used for long-term hydrological studies of other transboundary river basins in data-scarce regions like Iran.

Further research on the possible driving forces of deforestation and agricultural expansion found in this study is required to establish effective management strategies. For example, it may be necessary to determine the role of forest management institutions and socio-political drivers, and how and to what extent deforestation affects the livelihood of domestic end-users. It may also be necessary to examine how the impact of the political environment, and the income flow from Aras River Basin protection, influences the conversion of forest to agricultural land, and vice versa.

## Figures and Tables

**Table 1:** Specifications of Landsat images used in this study.

Data	Source	Date	Objective/Description
Digital elevation model (DEM)	ASTER	2014	Used for modeling/Spatial resolution of 30 m
Infrastructure maps	OSM database	2011	Used to produce LC maps and extract road layers and natural zones
	DIVA GIS	-	Include roads, rivers, political zones
	World watershed database	-	Zoning of basins
Landsat images	Landsat 5 TM	1983/07/10 1984/07/07 1984/07/23 1985/07/27	Used to produce LC maps Used to produce LC maps Used to produce LC maps Used to produce LC maps
	Landsat 7 ETM	2000/07/29 2000/07/22 2000/07/22 1999/06/13	Used to produce LC maps Used to produce LC maps Used to produce LC maps Used to produce LC maps
	Landsat 7 TM^+^	2010/07/10 2010/07/28 2010/07/28 2009/06/27	Used to produce LC maps Used to produce LC maps Used to produce LC maps Used to produce LC maps
	Landsat 8 OLI	2017/07/28 2017/07/28 2017/07/05 2017/06/03	Used to produce LC maps Used to produce LC maps Used to produce LC maps Used to produce LC maps
Google Earth images		Historical images	Accuracy assessment and correction

**Table 2:** Description of different scenarios identified based on the 1984–2000 calibration period used for defining scenarios for predicting LC changes.

Scenario Code	Scenario Name	Sub-Model	Input Variables	Prediction for Year	Reference Map for Validation
1	Forest to agriculture	A	DEM map Distance from bare land in 1984 Distance from agriculture in 1984 Distance from forest in 1984 Distance from residential areas in 1984 Distance from river in 1984	2010 2017 And 2027	Generated LC map in 2010 Generated LC map in 2027 None
2	Agriculture to rangeland	B	DEM map Distance from bare land in 1984 Distance from agriculture in 1984 Distance from forest in 1984 Qualitative variables in rangeland	2010 2017 And 2027	Generated LC map in 2010 Generated LC map in 2027 None
3	Bare land to rangeland	C	Distance from forest in 1984 Distance from agriculture in 1984 Distance from residential areas in 1984 Qualitative variables in rangeland	2010 2017 And 2027	Generated LC map in 2010 Generated LC map in 2027 None
4	Rangeland to agriculture	D	DEM map Distance from bare land in 1984 Distance from agriculture in 1984 Distance from forest in 1984 Distance from residential areas in 1984	2010 2017 And 2027	Generated LC map in 2010 Generated LC map in 2027 None
5	All transmission sub-models	E	DEM map Distance from river in 1984 Distance from agriculture in 1984 Distance from bare land in 1984 Distance from residential areas in 1984 Distance from rangeland in 1984 Distance from forest in 1984 Qualitative variables in all sub-models	2010 2017 And 2027	Generated LC map in 2010 Generated LC map in 2027 None

**Table 3:** Description of different scenarios identified based on the 2000–2010 calibration period used for defining scenarios for predicting future LC changes.

Scenario Code	Scenario Name	Sub-Model	Input Variables	Prediction for Year	Reference Map for Validation
1	Agriculture to forest	A	DEM map Distance from bare land in 2000 Distance from agriculture in 2000 Distance from forest in 2000 Qualitative variables in forest	2017 2027 And 2037	Generated LC map in 2017 None None
2	Agriculture to rangeland	B	DEM map Distance from bare land in 2000 Distance from agriculture in 2000 Distance from forest in 2000 Qualitative variables in rangeland	2017 2027 And 2037	Generated LC map in 2017 None None
3	forest to agriculture	C	DEM map Distance from bare land in 2000 Distance from agriculture in 2000 Distance from forest in 2000 Qualitative variables in agriculture	2017 2027 And 2037	Generated LC map in 2017 None None
4	Rangeland to agriculture	D	DEM map Distance from bare land in 2000 Distance from agriculture in 2000 Distance from forest in 2000 Qualitative variables in agriculture	2017 2027 And 2037	Generated LC map in 2017 None None
5	Rangeland to bare land	E	DEM map Distance from bare land in 2000 Distance from agriculture in 2000 Distance from forest in 2000 Qualitative variables in bare land	2017 2027 And 2037	Generated LC map in 2017 None None
6	All transmission sub-models	F	DEM map Distance from bare land in 2000 Distance from agriculture in 2000 Distance from forest in 2000 Distance from rangeland in 2000 Qualitative variables in all sub-models	2017 2027 And 2037	Generated LC map in 2017 None None

**Table 4:** Accuracy of the produced land cover (LC) maps based on the Kappa coefficient.

	Forest	Agriculture	Rangelands	Bare Lands	Deep Water	River	Residential	Overall Kappa
LC map 1984	0.80	0.75	0.82	0.92	0.96	0.83	0.99	0.87
LC map 2000	0.79	0.78	0.84	0.90	0.97	0.86	0.98	0.87
LC map 2010	0.83	0.72	0.79	0.88	0.94	0.81	0.96	0.85
LC map 2017	0.89	0.74	0.81	0.94	0.98	0.78	0.99	0.88

**Table 5:** Description of changes in water bodies from 1984 to 2017 in Aras River basin.

Number of Dams	Country	Coordinates (meters)	Area (hectares)	Construction Period	Description
1	Armenia	X:570211 Y: 4390210	724.5	1984–2000	Concrete da m built to store water
2	Armenia	X:5468b6 Y: 4396219	98.37	1984–2000	Embankment dam built to store water for irrigation
3	Nakhchivan	X: 543572 Y: 4359306	283	2000–2010	Concrete dam built to store water and generate power
4	Armenia	X: 479771 Y: 4402030	1403	1984–2017	Aquaculthre production
5	Armenia	X: 621165 Y: 4355374	17	1984–2000	Soil dam for water storage and infiltration
6	Armenia	X:62b851 Y: 4344088	299	1984–2017	Soil dam built for water storage and feed agriculture

**Table 6:** Cramer’s V coefficients for the driver variables of LC changes from 1984 to 2000.

Variables	DEM	Aspect	Slope	Distance from Rangeland	Distance from Roads	Distance from River	Distance from Agriculture	Distance from Bare Land	Diitance from Deep Water	Distance from Residential Areas
Overall Cramer’s V	0.214	0.131	0.129	0.171	0.023	0.034	0.164	0.129	0.197	0.231
Rangeland	0.182	0.0 67	0.154	0.213	0.078	0.046	0.324	0.289	0.138	0.074
Bare land	0.158	0.040	0.124	0.142	0.041	0.087	0.143	0.129	0.174	0.057
Agriculture	0.141	0.084	0.041	0.112	0.034	0.068	0.071	0.176	0.143	0.043
Residential areas	0.02	0.0 27	0.039	0.023	0.042	0.097	0.017	0.132	0.041	0.084
Rivers	0.174	0.128	0.302	0.228	0.038	0.047	0.238	0.154	0.487	0.045
Deep water	0.413	0.056	0.079	0.143	0.072	0.079	0.359	0.402	0.184	0.1 37
Forests	0.002	0.000	0.021	0.012	0.000	0.014	0.000	0.012	0.009	0.001
